# Fractures of the lateral malleolus – a retrospective before-and-after study of treatment and resource utilization following the implementation of a structured treatment algorithm

**DOI:** 10.1186/s12891-022-05358-x

**Published:** 2022-04-29

**Authors:** Emilia Möller Rydberg, Jonas Skoglund, Hampus Brezicka, Jan Ekelund, Mikael Sundfeldt, Michael Möller, David Wennergren

**Affiliations:** 1 Institute of Clinical Sciences, Sahlgrenska Academy, University of Gothenburg, Gothenburg, Sweden; 2 Department of Orthopaedics, Sahlgrenska University Hospital, Gothenburg/Mölndal, Sweden; 3 Centre of Registers Västra Götaland, Gothenburg/Mölndal, Sweden

**Keywords:** Ankle Fracture, Swedish Fracture Register, Fracture management, Treatment algorithm

## Abstract

**Background:**

In 2015 a study of isolated lateral malleolar fractures (AO/OTA44-B1) treated at Sahlgrenska University hospital (SU) during two consecutive years revealed large-scale variation in the choice of treatment and planned follow-up. The study resulted in the development of a structured treatment algorithm (TA) for ankle fractures. We investigated the effects of this well-implemented TA on the classification, treatment and follow-up of lateral malleolar fractures.

**Methods:**

The current study is an uncontrolled, non-randomized, retrospective before-and-after study comparing a group of AO/OTA44-B1 fractures treated at SU before the introduction of the TA for ankle fractures (1 April 2012 to 31 March 2014) with a group treated after the introduction of the TA (1 September 2017 to 31 August 2019).

**Results:**

In all the studied parameters regarding treatment for AO/OTA44-B1 fractures, a statistically significant change was seen after the introduction of the TA. Surgical treatment reduced from 32% (95% CI 27.5 – 36.5) pre-TA to 10% (95% CI 6.9 – 13.1) post-TA, while the number of patients permitted full weight-bearing increased from 41% (95% CI 36.3 – 45.7) to 84% (95% CI 80.1 – 87.9).

**Conclusions:**

A thoroughly implemented treatment algorithm can reduce the number of surgical treatments for stable ankle fractures. The current study demonstrates that a structured treatment algorithm can standardize the management of ankle fractures and make decisions less dependent on the surgeon’s discretion.

## Background

Ankle fractures are defined as fractures to one or more of the malleoli, combined or not, with injuries to adjacent ligaments. Transsyndesmotic lateral malleolar ankle fractures are considered stable or unstable in the ankle mortise, depending on the presence or absence of fracture or ligamentous injury to the medial aspect of the ankle (1). The AO/OTA classification considers both fractures and ligamentous injuries and thus reflects stability. Understanding whether an ankle fracture is stable or unstable in the ankle mortise is essential for further decision-making regarding management and treatment (2, 3).

The Swedish Fracture Register (SFR) is a national quality register of all types of fractures treated by orthopedic surgeons, both surgically and non-surgically (4). Since 2021, the SFR has had full coverage of all the orthopedic departments in Sweden. Fractures are registered in the SFR by the attending physician at patient presentation at the accident and emergency department (4). In the SFR, ankle fractures are classified according to the AO/OTA classification system. Juto et al. have reported substantial accuracy of ankle fracture classification in the SFR (5).

In 2015, a study was conducted at Sahlgrenska University Hospital (SU) presenting the epidemiology of all ankle fractures registered in the SFR during two consecutive years (April 2012-March 2014), as well as a detailed analysis of the management of lateral malleolar fractures classified as AO/OTA44-B1 during that period (6). The study revealed the difficulty in everyday practice in distinguishing whether a lateral malleolar fracture is isolated and stable or has an associated medial ligament injury, making it unstable. The study further demonstrated a large variation within the same department with regard to weight-bearing restrictions, choice of treatment and planned follow-up (6). The study resulted in the development of a structured treatment algorithm (TA) for ankle fractures at SU. Before the introduction of the TA at SU, no structured treatment algorithm had been in place for ankle fractures, leaving the decision regarding choice of treatment largely at the surgeon’s discretion. The aim of introducing a structured TA for ankle fracture management was to clarify the indications for surgical treatment. This was thought to reduce the number of operations and subsequent complications to surgery and optimize resource consumption (7). Another aim of the TA was to limit weight-bearing restrictions and reduce unnecessary radiographic examinations for stable isolated lateral malleolar fractures, as this has been shown not to be necessary (8, 9).

The aim of the current study was to evaluate the effect of a structured, well-implemented TA on the classification and treatment of lateral malleolar fractures.

## Methods

This study focuses on the fractures registered as AO/OTA44-B1 fractures before and after the introduction of the TA, but it will also briefly describe the epidemiology of all ankle fractures registered in the SFR at SU during the given timeframes.

The TA was introduced and thoroughly implemented at the department and was regarded as fully implemented in September 2017. The treatment algorithm included information on fracture classification, the choice of recommended treatment plan for each fracture group and plans for follow-up and recommended weight-bearing. The main features of the TA are displayed in Table 1 (Table 1).

**Table 1:** The main features of the TA with recommendations for each fracture group or subgroup

AO/OTA-group/subgroup	Stability	Treatmentplan	Weight-bearing	Follow-up	Radiographic examination
A1	Stable	Ankle brace	Full	4 w	None
B1.1		Ankle orthosis			
B1.2	Possibly unstable	Cast		6 w	One week
B1.3					
A2	Unstable	Surgery	Full	3 w	Postoperatively
A3					
B2					
B3			Partial weight-bearing for 3 weeks		
C1					
C2					
C3					

Regarding transsyndesmotic ankle fractures, the main objective of the TA was to clarify that lateral malleolar fractures with clinical signs of a deltoid ligament injury should be classified as AO/OTA44-B2 and treated surgically, whereas lateral malleolar fractures without signs of medial ligament injury should be classified as AO/OTA44-B1 and consequently treated non-surgically. Signs of medial ligament injury was defined as findings of tenderness over the deltoid ligament on initial physical examination as this was procedure advocated at the department at the time of the study.

**Fig. 1:**
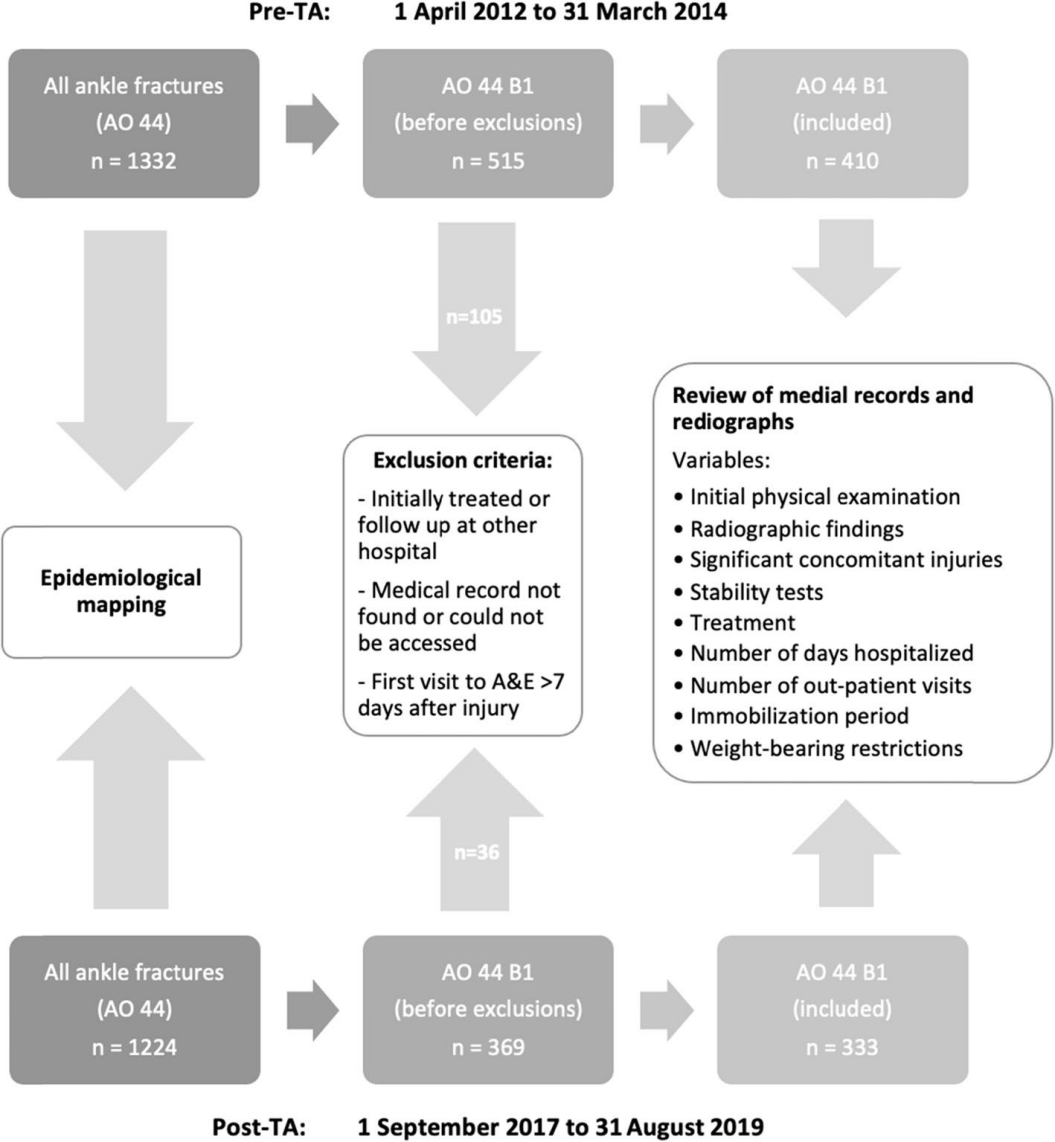
Flow chart showing how the study of AO/OTA44-B1 fractures was conducted

The current study is an uncontrolled, non-randomized, before-and-after study comparing a group of consecutive AO/OTA44-B1 fractures registered in the SFR at SU before the introduction of the TA (1 April 2012 to 31 March 2014) with a group registered after the introduction of the TA (1 September 2017 to 31 August 2019) (Fig. 1). The group of fractures occurring before the TA (April 2012 to March 2014) will be referred to as the pre-TA group and, consequently, the group of fractures registered after the introduction of the TA (September 2017 to August 2019) will be referred to as the post-TA group. The dataset for the pre-TA group is the same dataset as used for the study conducted in 2015 (6).

In order to be able to evaluate the effect of the TA, all patients seeking care for their ankle fracture more than 7 days after the injury were excluded from the study as the treatment algorithm was assessed as not being applicable that long after the trauma. Regarding the pre-TA group, a new dataset for the study period was extracted from the SFR, after which the same initial exclusions as in the study in 2015 were made and additional exclusions due to the patients not seeking medical care within 7 days from the injury (6).

Data from the SFR relating to fracture classification, age, sex, injury mechanism, frequency of high-/low-energy trauma and frequency of closed/open fracture were analyzed. For AO/OTA 44-B1 fractures, medical records and radiographs were reviewed, in addition to the data from the SFR. From the medical records, the following variables were studied: signs of medial ligament injuries found at the initial physical examination, instructions regarding weight-bearing restrictions, duration of immobilization period, number of outpatient visits, days hospitalized and number of radiographic examinations performed.

Treatment was reported as surgical, non-surgical or conversion of treatment from non-surgical to surgical at an early stage. For the analysis of treatment method, the cases in which non-surgical treatment was changed to surgical at an early stage were included in the group of surgically treated patients. For the individual cases, medical records were closely studied and described separately with regard to the reasoning behind the change of treatment plan.

The current study was approved by the Regional Ethical Review Board in Gothenburg, Sweden (reference number 1011–15). All patients were informed at registration in the Swedish Fracture Register that they had the right to withdraw. According to Swedish legislation, National Quality Registers do not require signed consent from the individual registered patient.

### Statistical methods

Continuous variables are presented as the mean (SD) and medians (range), while categorical data are presented using frequency and percentages. Statistical tests comparing demographic data between the pre-TA and the post-TA were performed using independent samples t-test and Fisher-s exact test. The distribution between AO/OTA fracture groups was compared between pre-TA and post-TA using the Chi-squared test. The effect of the TA was evaluated by comparing outcome variables between the pre-TA group and the post-TA group utilizing Fisher’s exact test and the Mann–Whitney U test.

The statistical software was IBM SPSS Statistics, Version 25.

## Results

### All ankle fractures

During the pre-TA study period, 1,328 patients with ankle fractures were identified, while four patients had sustained two ankle fractures, making the total number of fractures 1,332. During the post-TA study period, 1,217 patients with ankle fractures were identified, six of these patients had sustained bilateral ankle fractures and one patient had two ankle fractures during the two-year period, making the total number of fractures 1,224 (Fig. 1). In the pre-TA group the same 73 fractures were excluded as in the study in 2015 (due to initial treatment or follow-up conducted at another hospital or that the medical records could not be accessed) and an additional 32 patients were excluded due to not seeking care within 7 days after the injury. This resulted in a remaining total of 410 ankle fractures for final analysis. In the post-TA group, 369 fractures were registered as AO/OTA-B1 and, of these, 36 were excluded by the same exclusion criteria as in the pre-TA group, leaving a remaining 333 fractures in the post-TA group to be included in the final analysis (Fig. 1).

No clinically important differences were seen between the groups with regard to demographic data (Table 2). In the pre-TA group, the total mean age for all ankle fractures was 53 years (SD 20) and 58% were women. The corresponding number for the post-TA group reveals that the mean age was 54 years (SD 19) and that 63% were women (Table 2).

**Table 2:** Demographics of all ankle fractures (AO/OTA 44) registered in the SFR at Sahlgrenska University Hospital before (pre-TA) and after (post-TA) the introduction of the structured treatment algorithm. P-values provided for the differences between the Pre-TA and the Post-TA groups

	**Pre-TA** (*n* = 1332)	**Post-TA** (*n* = 1224)	***p*-value**
Mean age Total, years (SD) Male, years (SD) Female, years (SD)	53 (20) 47 (19) 57 (19)	54 (19) 49 (19) 57 (19)	0.103*
Sex Male, *n* (%) Female, *n* (%)	560 (42) 772 (58)	455 (37) 769 (63)	0.012**
Open fractures, *n* (%)	22 (1.7)	28 (2.3)	0.256**
High energy, *n* (%)	76 (5.7)	48 (3.9)	0.065**

^*^ Independent samples t-test

^**^Fisher’s exact test

The frequency of open fractures in the pre-TA and post-TA groups was 1.7% and 2.3% respectively. In the pre-TA group, 5.7% of the fractures were due to high-energy trauma, compared with 3.9% in the post-TA group (Table 2).

### Distribution between AO/OTA fracture types and groups

**Fig. 2:**
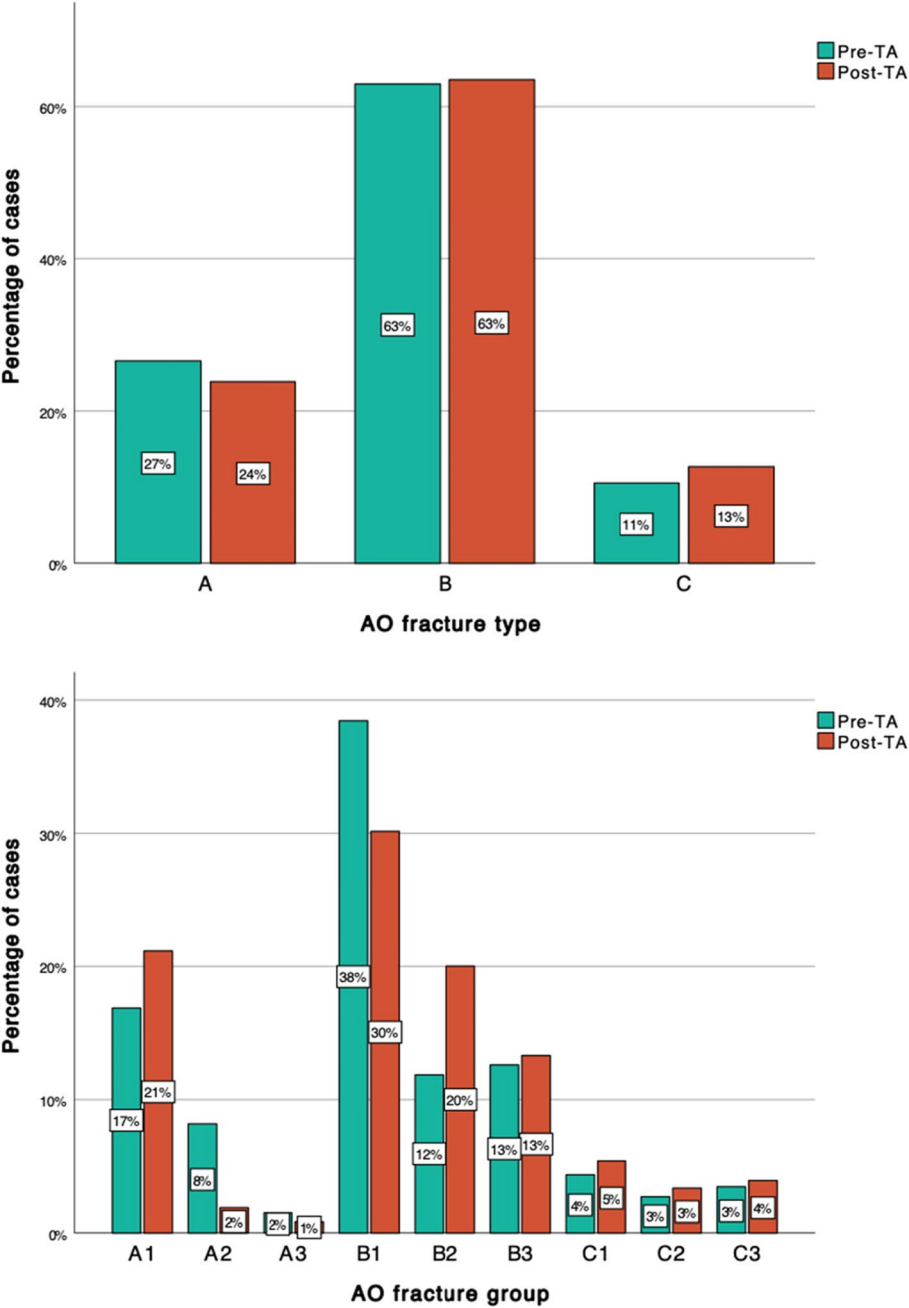
Distribution of fractures according to AO/OTA fracture type and group, before (pre-TA) and after (post-TA) the introduction of the structured treatment algorithm

The distribution between AO/OTA fracture types and groups is displayed in Fig. 2. It shows a similar distribution between fracture types (A, B and C) pre- and post-TA (p = 0.107). With regard to the distribution between fracture groups, a reduction in AO/OTA44-B1 frectures from 38 to 30% and a corresponding increase in B2 fractures from 12 to 20% was seen. Within the AO44A type fractures, an increase was seen in the A1 group, from 17% pre-TA to 21% post-TA. A corresponding reduction in A2 fractures was seen from 8% pre-TA to 2% post-TA (Fig. 2). The overall difference in distribution between AO/OTA fracture groups in the Pre-TA and the Post-TA groups was statistically significant (p < 0.001).

### Choice of treatment for each specific AO/OTA fracture group

A statistically significant reduction in surgically treated ankle fractures was seen at SU after the introduction of the treatment algorithm (p = 0.001). In the pre-TA group, 640/1,332 (48%) were treated surgically, compared with 505/1,224 (41%) in the post-TA group (Table 3).

**Table 3:** Treatment of ankle fractures by AO group, before and after the introduction of a structured treatment algorithm

	**Surgical treatment** *n* (%)	**Non-surgical treatment** *n* (%)	*p*-value* (treatment method)
All fractures Pre-TA Post-TA	640 (48) 505 (41)	692 (52) 719 (59)	0.001
A1 Pre-TA Post-TA	10 (4.4) 0 (0)	215 (96) 259 (100)	< 0.001
A2 Pre-TA Post-TA	51 (47) 13 (57)	58 (53) 10 (44)	0.493
A3 Pre-TA Post-TA	12 (60) 8 (80)	8 (40) 2 (20)	0.419
B1 Pre-TA Post-TA	158 (31) 36 (9.8)	354 (69) 333 (90)	< 0.001
B2 Pre-TA Post-TA	133 (84) 178 (73)	25 (16) 67 (27)	0.007
B3 Pre-TA Post-TA	160 (95) 151 (93)	8 (4.8) 12 (7.4)	0.362
C1 Pre-TA Post-TA	47 (81) 41 (62)	11 (19) 25 (38)	0.029
C2 Pre-TA Post-TA	34 (94) 36 (88)	2 (5.6) 5 (12)	0.438
C3 Pre-TA Post-TA	35 (76) 42 (88)	11 (24) 6 (13)	0.185

^*^Fisher’s exact test

A statistically significant reduction in surgically treated fractures was also seen in the individual AO/OTA groups AO44-A1, -B1, -B2 and -C1 (Table 3).

#### AO/OTA44-B1 fractures

In the closely studied group of AO/OTA44-B1fractures, no significant differences between the pre- and post-TA groups were seen with regard to demographic data (Table 4). The pre-TA and post-TA groups of AO/OTA-B1 fractures consisted of 221/410 (54%) and 195/333 (59%) women respectively. The mean age in the pre-TA group was 52 years (SD 19), while, in the post-TA group, the mean age was 53 years (SD19). None of the fractures in the post-TA group was open compared with 1.5% (n = 6) in the pre-TA group. The number of high-energy injuries were 3.4% (n = 14) and 2.7% (n = 9) in the pre-TA and the post-TA group respectively (Table 4).

**Table 4:** Demographics of AO/OTA44-B1 fractures registered in the SFR at Sahlgrenska University Hospital before (pre-TA) and after (post-TA) the introduction of the structured treatment algorithm. P-values provided for the differences between the Pre-TA and the Post-TA groups

	**Pre-TA** (*n* = 410)	**Post-TA** (*n* = 333)	***p*-value**
Mean age Total, years (SD) Male, years (SD) Female, years (SD)	52 (19) 48 (20) 56 (18)	53 (19) 52 (20) 53 (19)	0.735*
Sex Male, *n* (%) Female, *n* (%)	189 (46) 221 (54)	138 (41) 195 (59)	0.207**
Open fractures, *n* (%)	6 (1.5)	0 (0)	0.036**
High energy, *n* (%)	14 (3.4)	9 (2.7)	0.673**

^*^ Independent samples t-test

^**^Fisher’s exact test

### Findings of medial tenderness at clinical examination

As shown in Table 5, a statistically significant increase was seen in the number of patients in whom findings of medial tenderness at the first clinical examination were documented in the medical records, from 80% pre-TA to 86% post-TA (Table 5). A statistically significant reduction was also seen in the number of patients classified as AO/OTA44-B1 fractures, where medial tenderness was reported to be present at the first clinical examination, 49% pre-TA and 33% post-TA. Among the patients with no medial tenderness, a statistically significant increase in the proportion of non-surgical treatment was seen, from 86% pre-TA to 94% post-TA. In the group classified as AO/OTA44-B1 fractures but where medial tenderness was reported to be present in the medical records, an increase in the proportion of non-surgical treatment was also seen, from 47% pre-TA to 83% post-TA (Table 5).

**Table 5:** AO/OTA44-B1 fractures, treatment related to findings of medial tenderness at the first examination

	**Pre-TA** *n* (%)	**Post-TA** *n* (%)	*p*-value*
Medial tenderness commented on	328 (80)	288 (87)	0.024
Medial tenderness present	161 (49)	95 (33)	< 0.001
No medial tenderness Surgical treatment Non-surgical treatment	23 (14) 144 (86)	12 (6.2) 181 (94)	0.020
With medial tenderness Surgical treatment Non-surgical treatment	85 (53) 76 (47)	16 (17) 79 (83)	< 0.001

^*^Fisher’s exact test

### Treatment

In all the studied parameters regarding treatment (choice of treatment method, weight-bearing restrictions and immobilization time) for AO/OTA44-B1 fractures, a statistically significant change was seen after the introduction of the TA (Table 6). Surgically treated AO/OTA44-B1 fractures were reduced from 130/410 (32%) pre-TA to 34/333 (10%) post-TA (p < 0.001). The number of patients allowed full weight-bearing increased from 166/410 (41%) pre-TA to 278/333 (84%) post-TA (p < 0.001). Patients instructed to partially weight-bear were reduced from 181/410 (44%) to 22/333 (6.6%) after the introduction of the TA (p < 0.001). Regarding the time immobilized in a cast or brace, the mean time pre-TA was 45 days (SD 9) which was then reduced to 42 days (SD 10) post-TA (p < 0.001) (Table 6).

**Table 6:** Choice of treatment for AO/OTA44-B1 fractures before (pre-TA) and after (post-TA) the introduction of a structured treatment algorithm

	**Pre-TA** (*n* = 410)	**Post-TA** (*n* = 333)	***p*-value**
Surgically treated, *n* (%)	130 (32)	34 (10)	< 0.001*
Non-surgically treated, *n* (%)	280 (68)	299 (90)	
Full weight-bearing allowed, *n* (%)	166 (41)	278 (84)	< 0.001*
Partial weight-bearing allowed, *n* (%)	181 (44)	22 (6.6)	< 0.001*
Immobilization time, days Median (range) Mean (SD)	43 (14–108) 45 (8.99)	42.0 (0–95) 41.87 (10)	< 0.001**

^*^ Fisher’s exact test

^**^ Mann–Whitney U test

Of all the non-surgically treated patients, the treatment plan was converted to surgical treatment at an early stage in 5 patients (1%) in the pre-TA group and in 2 patients (0.6%) in the post-TA group. In three of the cases in the pre-TA group, a slight lateralization of talus at the one-week radiographic follow-up was found, after which the treatment plan was changed. In one patient, a stability test was performed in the operating theater, indicating instability in the ankle joint, and, in one case, no clear indication for a change of treatment plan was found in medical records or radiographic images. In the post-TA group, the two patients in whom the treatment was converted were found to have a slight increase in the displacement in the fracture at the one-week radiographic follow-up, after which the treatment plan was changed.

### Resource consumption

A statistically significant reduction in the number of radiographic examinations performed and the number of days hospitalized was seen for AO/OTA44-B1 fractures pre-TA compared with post-TA (p < 0.001) (Table 7). The number of outpatient visits was also reduced after the introduction of the treatment algorithm, but this reduction was not found to be statistically significant (Table 7).

**Table 7:** Resource consumption for AO/OTA44-B1 fractures before (pre-TA) and after (post-TA) the introduction of a structured treatment algorithm

	**Pre-TA** (*n* = 410)	**Post-TA** (*n* = 333)	*p*-value*
Number of radiographic examinations Median (range) Mean (SD)	2 (1–9) 2.5 (1)	2 (1–5) 2.2 (0.8)	< 0.001
Days hospitalized Median (range) Mean (SD)	0 (0–35) 1 (2.7)	0 (0–17) 0.7 (2.6)	< 0.001
Number of out-patient visits Median (range) Mean (SD)	3 (1–11) 3.5 (1.3)	3 (0–7) 3.2 (0.9)	0.055

^*^ Mann–Whitney U test

## Discussion

The most important finding in this study is that by implementing a structured treatment algorithm significantly fewer patients with a stable ankle fracture were surgically treated. This study further demonstrates that a treatment algorithm for ankle fractures can be implemented and used by a variety of doctors in everyday clinical practice at a large teaching hospital with good adherence.

The main objective of the treatment algorithm was to ensure that ankle fractures are classified, treated and followed up correctly. Another aim was to reduce the number of unnecessary surgeries and subsequent complications to surgery (10). The aim of the current study was to evaluate the effects of that treatment algorithm. The current study focuses on isolated transsyndesmotic lateral malleolar fractures without signs of deltoid ligament injury, AO/OTA44-B1. These fractures are defined as stable and can be safely treated non-surgically (11–14). The current study demonstrates that they received adequate treatment to a greater degree after the implementation of the TA, as a reduction was seen from 32 to 10% of patients with AO/OTA44-B1 fractures receiving surgical treatment.

In the current study, suspicion of a deltoid ligament injury was defined as findings of medial tenderness on the first clinical examination as this was the method used at the department at the time of the study. The best method for assessing deltoid ligament integrity remains unsolved (15). Medial tenderness, ecchymosis and swelling is by some seen as unreliable predictors of deltoid ligament injury (16–18). Magnetic resonance imaging (MRI) has been argued the best noninvasive method of evaluating the deltoid ligament, but has the disadvantage of high costs and often low availability (16, 19). A stress tests with measurement of the medial clear space is presented in some studies as the method of choice but in others as unreliable and associated with the risk of generating false positive results, exposing stable fractures to the risks of surgery (20–24).

A lateral malleolar fracture with medial ligament injury is by definition an AO/OTA-B2 fracture (25). This was one of the key messages regarding B fractures in the TA. As demonstrated in this study, AO/OTA44-B1 fractures classified correctly with respect to medial tenderness increased significantly after the introduction of the TA, as AO/OTA44-B1 fractures with documented medial tenderness were reduced from 49 to 33%. This study further demonstrates that “true” B1 fractures (with no medial tenderness) that are subject of surgical treatment were reduced significantly from 14 to 6%. Our interpretation of this is that a better understanding of the AO/OTA classification of ankle fractures was achieved by implementing the TA, together with an enhanced consideration of indications for surgery and a reduction in surgical treatment for stable fractures.

Numerous studies have reported the benefits of early full weight-bearing and early range of motion exercises for ankle fractures (26–29). The current study shows that the proportion of patients with AO44-B1 fractures that were allowed to fully weight-bear increased and the number of days immobilized in a cast was reduced after the introduction of the TA.

After the introduction of the structured TA for all ankle fractures, a shift in classification was seen between the AO/OTA groups. The proportion of fractures classified as B1 was reduced and the group of B2 fractures increased. This could be due to more fractures being correctly classified as B2, when the clinical examination indicated adjacent ligamentous injuries to the medial side of the ankle, as mentioned, one of the key messages in the TA. The finding that fewer AO/OTA444-B1 fractures were reported to have medial tenderness at the first clinical examination supports this assumption. However, the finding that the proportion of B2 fractures receiving surgical treatment was reduced raises a suspicion that stable fractures might have been mis-classified as AO/OTA44-B2 in this group. To elucidate this, a review of medical records for all AO/OTA44-B2 fractures would have had to be made.

The finding that the proportion of fractures receiving surgical treatment was reduced significantly in both the AO/OTA44-B1 and B2 groups demonstrates that fractures were not simply moved between AO/OTA groups and continued receiving surgical treatment, but that an actual overall reduction in the surgical treatment of ankle fractures was seen. Despite this, in only two cases in the post-TA group was non-surgical treatment changed at an early stage to surgical treatment compared with five patients in the pre-TA group. This could be due to the TA not advocating follow-up visits with radiographic examinations for B1 fractures. However, investigating whether there has been any increase in complications such as malunion, non-union or post-traumatic arthrosis was not within the scope of this study and this remains to be elucidated in future studies.

Another aim of the treatment algorithm was to optimize resource consumption. As the results of the current study demonstrate, the number of outpatient visits and radiographic examinations, as well as days hospitalized, was reduced by implementing a structured algorithm for follow-up.

The findings in this study are in line with the findings of Palm et al., demonstrating the great value of introducing treatment algorithms in orthopedic surgery (30). As demonstrated by Jain et al., and again in the current study, significant improvements in the management and optimization of resource consumption can be achieved for ankle fractures by educating colleagues and implementing guidelines (12).

One limitation to this study is the lack of long-term follow-up and patient reported outcome measures. The study demonstrates a reduction in surgically treated ankle fractures, but it remains unclear whether a subsequent reduction in postoperative complications and reoperations will follow. Future studies will investigate this and display how the reported changes have affected patient reported recovery. Further, as mentioned above, a long-term study of the AO/OTA44-B fractures that receive non-surgical treatment without a radiographic follow-up would be of great value. Another limitation is the fact that the current study evaluates the effects of the TA by comparing outcome measurements before and after the introduction of the TA, but it does not consider other factors that might change over time and influence the same outcome measurements. However, the study is a single center study over a limited timeframe where no other, to us known, changes were made at the same time.

One of the strengths of this study is the thorough review of 743 medical records that was performed. This study will be generalizable because it was conducted at a large orthopedic department, showing significant results for fractures that are common worldwide and treated every day.

## Conclusions

A thoroughly implemented treatment algorithm can reduce the number of surgical treatments for stable ankle fractures. The current study demonstrates that a structured treatment algorithm can standardize the management of ankle fractures and make decisions less dependent on the surgeon’s discretion.

## Data Availability

The datasets used and analysed during the current study are available from the corresponding author on reasonable request.

## Abbreviations

SU: Sahlgrenska University hospital
TA: Treatment algorithm
AO: Arbeitsgemeinschaft für Osteosynthesefragen
OTA: Orthopaedic Trauma Association
SFR: The Swedish Fracture Register
MRI: Magnetic resonance imaging
